# Clinical application of combined CRP and PCT detection in diagnosing and prognosing fracture-related infections

**DOI:** 10.3389/fcimb.2025.1563170

**Published:** 2025-03-14

**Authors:** Xiaojie Liu, Jie Lian, Fei Liu, Dunxin Han, Chenglin Sang

**Affiliations:** ^1^ Department of Orthopedics, 970 Hospital of the People's Liberation Army (PLA) Joint Logistic SupportForce, Weihai, Shandong, China; ^2^ Department of Orthopedics, 960 Hospital of the People's Liberation Army (PLA) Joint Logistic SupportForce, Jinan, Shandong, China

**Keywords:** fracture-related infections, procalcitonin, C-reactive protein, diagnostic value, sensitivity and specificity

## Abstract

**Objective:**

To investigate the clinical significance of combined procalcitonin (PCT) and C-reactive protein (CRP) testing in the diagnosis of fracture-related infections (FRIs).

**Methods:**

A retrospective analysis was performed on 252 patients with bone infections admitted between January 2018 and September 2024. CRP and PCT levels were measured in all patients both at diagnosis and postoperatively. The sensitivity, specificity, positive predictive value (PPV), and negative predictive value (NPV) of combined CRP and PCT for diagnosing FRIs were calculated. Based on clinical follow-up data, patients were divided into low-level and high-level groups according to the changes in CRP and PCT levels, and clinical outcomes, including length of hospital stay and infection control, were analyzed.

**Results:**

The postoperative levels of CRP and PCT in the infection group were significantly higher than in the control group (P < 0.05). The sensitivity, specificity, PPV, and NPV of combined CRP and PCT detection were 90.48%, 96.83%, 96.61%, and 91.04%, respectively. Patients in the high CRP and PCT group had a significantly longer hospital stay compared to the low-level group, and infection control rates were lower. Combined CRP and PCT detection was more effective in diagnosing and predicting clinical outcomes.

**Conclusion:**

Combined detection of CRP and PCT has high clinical application value in the diagnosis and prognosis evaluation of fracture-related infections, providing more accurate guidance, particularly in predicting recovery and infection control.

## Introduction

FRIs pose significant challenges, as these infections can lead to loss of limb function or even amputation (Depypere et al., 2020a). Treatment often involves multiple surgeries and prolonged hospitalizations, which substantially increase healthcare costs, causing immense suffering for patients and imposing significant economic and caregiving burdens on their families (Onsea et al., 2022; Prada et al., 2022). However, in the absence of typical clinical signs such as sinus tracts or purulence, FRIs are often diagnosed intraoperatively or postoperatively through findings such as intraoperative evidence of infection, bacterial cultures, or biochemical analysis (Depypere et al., 2020b; Rodarte et al., 2024). This diagnostic delay increases the risk of misdiagnosis, potentially compromising treatment outcomes.

The diagnosis of FRIs has always been a significant challenge in clinical practice, primarily because its symptoms often resemble those of other types of inflammatory responses and there is a lack of specific clinical manifestations. Early diagnosis of FRIs is particularly difficult in the absence of typical infection signs such as purulent discharge or sinus tracts. Traditional diagnostic methods, such as bacterial culture and imaging tests, although valuable in some cases, still have limited sensitivity and specificity and can be influenced by various factors, including antibiotic use, infection site, and the patient’s immune status. Furthermore, the delay in bacterial culture results and the diagnostic limitations of imaging tests further complicate the early diagnosis of FRIs. Therefore, the combined detection of biomarkers has become an effective strategy to improve diagnostic accuracy. Diagnosing FRIs is inherently complex and requires a multidisciplinary approach. Many current diagnostic protocols are adapted from algorithms designed for periprosthetic joint infections (Kraus et al., 2024), but data specific to FRIs remain limited. Laboratory tests play a crucial role in the diagnosis of infectious diseases. However, the symptoms and signs of acute infections caused by many bacteria or viruses are very similar. Additionally, there are numerous laboratory indicators related to infectious diseases, making it challenging for clinicians to choose the appropriate tests and determine the sensitivity of diagnostic markers. To establish early diagnostic categories for infectious diseases, quantify risk, monitor the progression and treatment of infections, and improve the effectiveness of distinguishing between bacterial and viral infections, we have selected easily detectable indicators in the blood for evaluation. The evaluation of inflammatory biomarkers in serum is an essential aspect of early diagnosis, offering advantages such as non-invasiveness, cost-effectiveness, and rapidity. Recent studies have explored novel serological diagnostic approaches, emphasizing the combination of multiple biomarkers, developing risk prediction models, and identifying potential infection-specific markers.

CRP, a commonly used marker for infectious diseases, is a nonspecific inflammatory biomarker (Wang et al., 2021). Following pathogenic invasion, CRP is synthesized in the liver to activate the immune system and facilitate pathogen clearance. Research by Sigmund et al. (2020) indicates that CRP may help distinguish between FRIs caused by high-virulence and low-virulence pathogens. PCT, known for its high sensitivity and specificity, offers the advantages of rapid and early detection, making it a promising biomarker for bacterial infections (Yang et al., 2023).Studies on the combined detection of CRP and PCT in other types of infections have shown that the two biomarkers can effectively improve the early diagnosis and prognostic assessment of bacterial infections. In pneumonia, research indicates that the combined detection of CRP and PCT enhances the sensitivity and specificity for diagnosing bacterial pneumonia. In the diagnosis of sepsis, the combined use of CRP and PCT is more advantageous than the use of either marker alone, as it enables early identification of bacterial infections and helps in assessing clinical prognosis. Although the combined detection of these two biomarkers has shown advantages in the diagnosis and prognosis of other infections, whether they can be used together for the diagnosis and clinical prognosis evaluation of bone infections remains inconclusive. This study aims to investigate the diagnostic value of combined CRP and PCT testing for the early and accurate identification of FRIs.

## 2 Methods

### 2.1 Patients

This study is a retrospective cohort study, selecting 252 patients with bone infections hospitalized between January 2018 and September 2024, with ages ranging from 18 to 85 years. All patients were diagnosed with bone infections confirmed by X-ray, CT, or MRI imaging and had received anti-infective treatment. Exclusion criteria included patients with malignant tumors, immune deficiencies, or those who did not complete follow-up. In this study, handling missing data is a crucial step to ensure the reliability of the results. To minimize the impact of missing data on the analysis, we can employ Multiple Imputation (MI) as the imputation method. MI generates multiple complete datasets by predicting and filling in the missing data several times, and then analyzes these datasets to combine the results. This approach enhances the precision of estimates and reduces bias. Additionally, using this method helps avoid the potential bias introduced by single imputation methods, ensuring the robustness and reliability of the final analysis. During the data analysis process, we will assess the pattern of missing data to ensure the applicability of the imputation method and conduct sensitivity analyses to validate the impact of imputation on the study conclusions. Through this rigorous data handling approach, we can enhance the credibility of the study results and ensure the validity of the conclusions.

### 2.2 Clinical data collection and grouping

Basic patient information, including age, sex, BMI, underlying diseases, etc., was collected, and the initial and postoperative CRP and PCT values were recorded. All patients underwent serological testing at admission, as well as on days 3, 7, and prior to discharge after treatment, to measure CRP and PCT levels. Among the included patients, 126 who developed FRIs during surgery were randomly assigned to the observation group, and another 126 non-infected patients were randomly selected as the control group. Based on the initial values of CRP and PCT, two criteria were established for high and low levels. High CRP was defined as CRP > 10 mg/L, and high PCT as PCT > 2 ng/mL. All patients were followed up regularly for 6 months after treatment to record hospitalization duration and infection control status. The study followed the Declaration of Helsinki, and all participants signed informed consent. The research protocol was approved by the Ethics Committee of the 960 Hospital.

### 2.3 Statistical analysis

All data were statistically analyzed using SPSS 22.0 software. Measurement data were expressed as mean ± standard deviation ( $\bar{\text{x}}$ ± s), and inter-group comparisons were performed using independent t-tests. Categorical variables were analyzed using chi-square tests. Multivariate logistic regression was used to assess the impact of combined CRP and PCT levels on patient prognosis. A P-value of <0.05 was considered statistically significant.

## 3 Results

### 3.1 Baseline characteristics

Among the 252 patients included, the average age was 57.3 ± 12.4 years, with 108 males (42.9%) and 144 females (57.1%). Among the patients, 25.4% had hypertension, 24.3% had diabetes, and 16.3% had chronic kidney disease. The average CRP and PCT levels at admission were 25.5 ± 12.8 mg/L and 1.2 ± 0.8 ng/mL, respectively (**Table 1**).

**Table 1:** Patient baseline characteristics.

Variables	N (percent)
CRP (-/+)	107 (42.5%)/145 (57.5%)
PCT (-/+)	119 (47.2%)/133 (52.8%)
Age (<60/≥60 y)	150 (59.5%)/102 (40.5%)
Gender (male/female)	134 (53.2%)/118 (46.8%)
BMI (<24/≥24)	98 (38.9%)/154 (61.1%)
Hypertension (absent/present)	64 (25.4%)/188 (74.6%)
Diabetes (absent/present)	62 (24.3%)/190 (75.7%)
Chronic kidney disease (absent/present)	41 (16.3%)/211 (83.7%)
Features of injury (open/closed/unavailable)	109 (43.4%)/98 (38.9%)/45 (17.8%)
Infection side distribution (left/right/bilateral)	115 (45.6%)/105 (41.7%)/32 (12.7%)

### 3.2 CRP and PCT levels effectively diagnose bone infections

There were no significant differences between the observation and control groups in terms of gender distribution, average age, average disease duration, fracture types, or preoperative PCT and CRP levels (P > 0.05). However, CRP and PCT levels in the infection group were significantly higher than in the control group on postoperative days 1, 2, 3, and 7 (P < 0.05). On postoperative days 1 to 3, CRP and PCT levels in both the FRIs group and control group showed an initial increase, and then gradually decreased, likely due to antibiotic use. In the control group, both markers exceeded normal values on postoperative day 1 but gradually returned to normal by day 7 (**Table 2**). Using a PCT > 2.0 ng/L threshold for diagnosis, the combined sensitivity and specificity for diagnosing FRIs were 90.48% and 96.83%, respectively, showing high diagnostic value (**Table 3**).

**Table 2:** Comparison of postoperative diagnostic markers.

Testing time	CRP		PCT	
	Control	FRIs	Control	FRIs
D1	6.62 ± 1.56	7.92 ± 0.99^*^	0.49 ± 0.28	0.66 ± 0.36^*^
D2	10.53 ± 3.48	16.92 ± 2.95^***^	1.23 ± 0.51	1.86 ± 0.61^**^
D3	8.92 ± 4.12	18.96 ± 4.33^***^	0.92 ± 0.49	3.15 ± 1.12^**^
D7	6.67 ± 3.15	10.92 ± 3.96^***^	0.76 ± 0.23	2.29 ± 0.49^***^

**Table 3:** Diagnostic performance of CRP and PCT.

Indicator	Positive patients	Negative patients	Total
CRP+ PCT+	114	4	118
CRP+ PCT-	12	122	134
Total	126	126	252

### 2.3 CRP and PCT levels predict clinical outcomes of bone infection

Based on CRP and PCT levels, patients were divided into four groups: high CRP, high PCT (CRP+PCT+), high CRP, low PCT (CRP+PCT-), low CRP, high PCT (CRP-PCT+), and low CRP, low PCT (CRP-PCT-). Results showed that the hospital stay of the CRP+PCT+ group (22.5 ± 3.4 days) was significantly longer than that of the CRP-PCT- group (15.8 ± 5.1 days). The infection control rate in the CRP+PCT+ group (60.3% ± 6.9) was significantly lower than that in the CRP-PCT- group (87.1% ± 7.8%) (**Table 4**). Furthermore, multivariate regression analysis showed that high CRP (HR=2.239, 95% CI = 1.320–3.978, p=0.033) and high PCT levels (HR=1.936, 95% CI = 0.983–3.593, p=0.041) were independent factors predicting hospitalization duration for bone-related infections (**Table 5**). Similarly, high CRP (HR=2.157, 95% CI = 1.101–4.897, p=0.045) and high PCT (HR=1.943, 95% CI = 0.914–3.785, p=0.029) were independent factors predicting infection control rates (**Table 6**).

**Table 4:** Patient stratification based on CRP and PCT levels and their clinical outcomes.

Group	Mean LHS (d)	Mean ICR (%)
CRP+ PCT+	22.5 ± 3.4	60.3 ± 6.9
CRP+ PCT-	19.2 ± 4.5	63.5 ± 8.3
CRP- PCT+	20.8 ± 6.8	70.7 ± 9.0
CRP- PCT-	15.8 ± 5.1	87.1 ± 7.8

LHS, Length of hospital stay.

ICR, Infection control rate.

**Table 5:** COX analysis of LHS.

Factors	Mean LHS (d)	Univariate			Multivariate		
		HR	95%CI	*P value*	HR	95%CI	*P value*
CRP ^-^/CRP ^+^	12.63/20.83	1.000/2.015	1.103–3.521	0.019^*^	1.000/2.239	1.320–3.978	0.033^*^
PCT ^-^/PCT ^+^	16.45/23.63	1.000/1.837	1.081–3.698	0.026^*^	1.000/1.936	0.983–3.593	0.041^*^
Age (<60/≥60y)	18.29/24.38	1.000/1.675	0.897–3.215	0.091			
Gender (female/male)	15.93/17.32	1.000/0.731	0.321–1.302	0.353			
BMI (<24/≥24)	20.66/17.93	1.000/0.704	0.402–1.329	0.245			

*p<0.05.

**Table 6:** COX analysis of ICR.

Factors	Mean ICR (%)	Univariate			Multivariate		
		HR	95%CI	*P value*	HR	95%CI	*P value*
CRP ^-^/CRP ^+^	49.74/61.89	1.000/2.568	1.207–4.582	0.032^*^	1.000/2.157	1.101–4.897	0.045^*^
PCT ^-^/PCT ^+^	52.37/70.43	1.000/1.897	1.017–3.867	0.011^*^	1.000/1.943	0.914–3.785	0.029^*^
Age (<60/≥60y)	48.837/59.623	1.000/1.685	0.987–3.348	0.683			
Gender (female/male)	61.78/53.39	1.000/0.564	0.348–1.841	0.286			
BMI (<24/≥24)	54.985/66.717	1.000/0.684	0.358–1.326	0.518			

*p<0.05.

## 4 Discussion and future perspectives

CRP is an acute-phase protein secreted by the liver in response to trauma, infection, or inflammation, triggered by pro-inflammatory cytokines. While CRP levels may increase due to surgical interventions, acute rejection, or other factors, relying solely on CRP for diagnosis is insufficient; it must be combined with comprehensive clinical evaluation. Advances in immunoturbidimetry have significantly improved CRP testing sensitivity (Bommenahalli Gowda et al., 2021). CRP levels can increase up to 1000-fold in inflammatory or infectious responses and can be easily detected in serum, returning to baseline after recovery (Coye et al., 2023). PCT, a glycoprotein precursor to calcitonin composed of 116 amino acids (Li et al., 2021), is secreted by the thyroid C-cells under normal physiological conditions but increases significantly in bacterial infections (Matur et al., 2022; Munsell et al., 2023). Consistent with this, in this study, PCT levels in the infection group were significantly higher than in the non-infected group (P < 0.05). Unlike CRP, PCT levels are usually unaffected by non-infectious factors, making it superior to CRP in differentiating bacterial infections from non-bacterial infections. It has higher diagnostic value in orthopedic infectious diseases and is considered an indispensable marker for diagnosing severe infections (Norman-Bruce et al., 2024). Additionally, PCT’s high sensitivity makes it an important tool for guiding antibiotic therapy (Schuetz, 2023). While CRP and PCT are widely used in various inflammatory diseases, literature on their combined use for predicting and diagnosing FRIs in orthopedic patients remains limited. This study aimed to assess the potential of combined CRP and PCT testing as biomarkers for diagnosing and prognostic evaluation of FRIs.

This study found that CRP levels on days 1, 2, 3, and 7 post-surgeries were significantly higher in the FRIs group than in the non-infected group (P < 0.05), indicating a significant elevation of CRP in infection responses. Previous literature has reported CRP sensitivity ranging from 68% to 90% and specificity from 71% to 88% (Klim et al., 2018; Zhang et al., 2023). In contrast, the combined sensitivity and specificity of CRP and PCT in this study were 90.48% and 96.83%, indicating higher diagnostic accuracy and reliability. Moreover, patients in the CRP+PCT group had a significantly longer hospital stay (22.5 ± 3.4 days) compared to those in the CRP-PCT group (15.8 ± 5.1 days). The infection control rate in the CRP+PCT group was significantly lower (60.3% ± 6.9%) than in the CRP-PCT group (87.1% ± 7.8%). Furthermore, both univariate and multivariate regression analyses showed that high CRP and PCT levels were important independent factors predicting clinical outcomes for bone infections. Furthermore, in addition to length of hospital stay and infection control, we could consider incorporating clinical outcome indicators such as mortality and long-term functional recovery into future studies to further assess the prognostic value of combined testing.

PCT and CRP are closely related to disease activity and can effectively indicate the type of infection. The diagnostic value of PCT and CRP levels in infectious diseases is higher than that of white blood cell count (WBC) levels, and their combined evaluation holds greater clinical significance. The combined application of PCT, CRP, and WBC can serve as an effective marker to distinguish between acute bacterial infections and non-bacterial infections (Li et al., 2021). Similarly, a study by Zheng et al. indicated that sTREM-1, PCT, and CRP have high predictive value for lung infections in patients with multiple trauma and ARDS (Zheng and Zhang, 2022). Ma’s research showed that levels of PCT, CRP, and FIB in bacterial pneumonia patients were higher than those in viral pneumonia patients. These biochemical markers can serve as independent predictors for diagnosing bacterial pneumonia and have high diagnostic value. The combined detection of these three markers provides the highest diagnostic efficiency and helps in the early clinical differential diagnosis of pneumonia infection types (Ma et al., 2023). CRP is more sensitive in capturing early inflammatory responses, while the specificity of PCT helps determine the presence of bacterial infections, thereby allowing for a more accurate assessment of the type and severity of the infection (Omaggio et al., 2024). Through this complementary mechanism, the combined testing of CRP and PCT not only improves the diagnostic accuracy for fracture-related infections but also effectively predicts clinical outcomes, providing a more reliable basis for personalized treatment.

However, this study also has some limitations. To control potential bias and improve the accuracy of the study results, this research employed strict inclusion and exclusion criteria to ensure the sample’s representativeness. All included patients were required to meet the radiological diagnostic criteria for bone infection, and special populations that could potentially affect the results, such as those with immune deficiencies or malignancies, were excluded. Additionally, to minimize the impact of infection severity on the results, patients were divided into high and low-level groups based on changes in CRP and PCT levels. Clinical outcomes such as hospital stay duration and infection control were analyzed to explore the predictive capacity of combined testing for fracture-related infections. Through these grouping methods, the clinical value of CRP and PCT combined testing in different infection severity levels could be more precisely assessed, avoiding undue influence from patients with severe infections. However, potential bias still exists in the selection of patients for CRP and PCT, and confounding factors such as antibiotic use and the severity of fracture-related infections were not considered in this study. We will attempt to avoid these issues in future research. Overall, the combined detection of CRP and PCT demonstrates high sensitivity, specificity, positive predictive value, and negative predictive value in the diagnosis of FRIs. This combined test provides an effective tool for the early diagnosis of FRIs in post-surgical orthopedic patients. Positive results from combined CRP and PCT testing provide strong evidence for the rational use of antibiotics and timely adjustments to treatment strategies. CRP and PCT, as commonly used infection markers, have broad clinical applications. Previous studies have primarily focused on their predictive role for infections in isolation. However, the course of bone infections is complex, and inflammation is often intense. A single indicator may not have sufficient sensitivity and specificity to fully reflect the condition. The combined detection of CRP and PCT in the clinical application of fracture-related infections can improve diagnostic accuracy, helping doctors more precisely assess the nature and severity of the infection. When both markers are significantly elevated, it suggests the presence of a severe bacterial infection, requiring timely adjustment of antibiotic treatment. If PCT is elevated while CRP remains low, it confirms a bacterial infection and guides treatment decisions. Combined testing can also be used for postoperative monitoring, assisting in the evaluation of infection control effectiveness and ensuring the individualized and precise adjustment of treatment plans.

## Data Availability

The original contributions presented in the study are included in the article/supplementary material. Further inquiries can be directed to the corresponding authors.
